# Social impact of a small-scale sporting event: the case of GIMNASTRADA 2019 in a world Heritage city

**DOI:** 10.3389/fspor.2026.1806965

**Published:** 2026-05-15

**Authors:** Vicente Luis-del Campo, Jesús Muñoz-Jiménez, Héctor Valentín Jiménez-Naranjo, Kiko Francisco León-Guzmán

**Affiliations:** 1 Faculty of Sports Sciences, University of Extremadura, Cáceres, Spain; 2 Faculty of Business, Finance and Tourism, University of Extremadura, Cáceres, Spain

**Keywords:** gymnastics, recreation, social impact, sporting event, UNESCO

## Abstract

**Introduction:**

The GIMNASTRADA is a recreational gymnastics event held every year in Cáceres (Spain), a UNESCO World Heritage Site since 1986. The first edition was held in 1996 and the last edition to date was in 2025. It has been held every year without interruption except for the 2020 edition, which was suspended due to COVID-19. The 2019 edition (23rd edition) was the most popular, with around 3,700 gymnasts and 12,000 spectators taking part. The aim of this study was to ascertain the social impact of GIMNASTRADA 2019 among residents and non-residents of Cáceres.

**Methods:**

To do this, a total of 210 participants anonymously answered a questionnaire about its possible positive effects (e.g., recognition and promotion of the city, fostering social and cultural exchange) and negative effects (e.g., increased traffic and insecurity, deterioration of the city and its surroundings). The questionnaire was answered via mobile devices using a Google Docs form and sent to the research team for further analysis.

**Results:**

Residents and non-residents scored similarly to the 12 answers of the questionnaire, except for the item related to the pride that the sporting event was held in the city. The results showed that residents of the host City (Cáceres) were more proud that the GIMNASTRADA was taking place in their city than non-residents (*p* < .001).

**Discussion:**

It is recommended that these results be taken into account in the management of future editions of this gymnastics event in order to reinforce the positive social impacts on the host city and limit the negative effects on both residents and foreign participants.

## Introduction

The GIMNASTRADA is a gymnastics-based sporting event with a playful and recreational character, which has taken place in the city of Cáceres since 1996. Consequently, it is framed within the “Gymnastics for All” philosophy, which is promoted by the International Gymnastics Federation (FIG). Unlike traditional competitive gymnastics, the GIMNASTRADA does not seek technical excellence or podium outcomes, but rather inclusive participation, choreographic creativity, and the enjoyment of physical activity. The most recent edition took place in the year 2025. Students from various educational centers in the city and the Autonomous Community of Extremadura participate in this gymnastic event, as well as participants from different Spanish provinces and regions of neighboring Portugal. It takes place in the covered sports pavilion of Cáceres, which has the capacity to host approximately 6,500 participants and is publicly owned (property of the Regional Government of Extremadura). Participation data corresponding to the 2019 edition of GIMNASTRADA were the highest of all editions held to date (3,700 gymnasts and 12,000 spectators). With these participation figures, the celebration of GIMNASTRADA implied a high density of occupation of urban space in a small city (96,000 inhabitants) that is also a UNESCO World Heritage City. Therefore, it constitutes an ideal case study for analyzing the social impacts of a sporting event in a historic city without compromising its sustainability. Furthermore, it turns GIMNASTRADA into a suitable social laboratory to analyze how “*bottom-up*” events (born from the educational/university base) can generate social impacts comparable or superior, in relative terms, to other “*top-down*” sporting events imposed by large international organizations.

GIMNASTRADA could be contextualized within sporting tourism since tourists travel to actively participate in or observe the sporting event, which usually also offers complementary activities with great attendance success (1). According to Gratton et al. (53), GIMNASTRADA 2019 would be a Type D sporting event, as it generates limited economic activity and participants mainly originate from the host country. Consequently, it would be treated as a small-scale sporting event, yet one that represents a source of wealth for the hosting communities due to the arrival of participants and companions, contributing to their social and economic development (54, 55). These small sporting events have not been as extensively studied in scientific literature due to their low economic impact (56). However, these small tourism products could generate a more sustainable image than a large sporting event because they utilize existing infrastructure (2).

The impacts of sporting events are varied; for instance, they include economic, social, tourism, cultural, psychological, and political impacts (3). In this vein, Parra-Camacho et al. (4) performed a study for scale validation to measure residents' perceptions about negative impacts of a sporting event, resulting in a multidimensional scale with four factors related to social, political and administrative, environmental, and socio-cultural impacts. While it is true that studies on economic and tourism impacts have been more abundant in scientific literature, social impacts are becoming increasingly interesting to researchers, policymakers, and event organizers because they partially justify the investment made in the event (5, 6). The methodology used by scientific literature to measure the social impact of a sporting event has been the use of different scales quantifying the perceptions of active participants (athletes) and passive participants (spectators) regarding various social issues related to the sporting event. Examples include cultural exchange, social problems, economic benefits, natural resources, traffic congestion, and pollution (7), or even the opportunity to gather with family and friends, meet new people, or become an interesting leisure option (8).

Currently, there is a lack of standardized approaches to address the social impact of a given event, whether sporting or not (57). However, we selected the Social Exchange Theory (SET) because it is a comprehensive framework claiming that human relationships are formed by the individual lens of the cost-benefit analysis (i.e., how human beings weigh rewards and costs that occur in their social interactions; see (9). We argue that SET would allow us to ascertain the impact of different variables on residents' perceptions (i.e., how they interpret the benefits and costs of the tourism impacts; see (10) and, more specifically, the support of the residents of the host city towards the sporting event (11). Within this rationale, the literature has reported a relation between residents’ satisfaction with quality of life and attitude to support sporting events (11, 12). This positive relationship found in the residents of the host city between their support to sporting events and their perception of quality of life has been also meditated by the size of the sporting event, being this support higher for small sporting events compared to mega-events (13).

At a social level, the social perception of residents regarding mega-sporting events has been studied in motor Grand Prix events (motorcycle or car), International Championships (European, World), or Olympic Games. For example, Waitt (14) found an increase of enthusiasm between Australian residents over the two-year period, the desire to bid for another Olympics, the degree of willingness to participate as a volunteer or feeling a sense of community inspired by the Olympic spirit. Ohmann et al. (15) concluded that residents of Munich perceived primarily positive impacts as a consequence of hosting the 2006 FIFA World Cup in Germany (e.g., urban regeneration, greater sense of security, positive fan behavior). Zhou (16) showed that most respondents perceived positively the social impacts of hosting the Macau Formula-3 Grand Prix, being the age, attitude towards government performance, and interest to increase tourists the most relevant factors influencing their perceptions. Additionally, Prayag et al. (17) indicated that economic and socio-cultural impacts were related to the overall attitude of residents for the 2012 Olympic Games. Gibson et al. (18) found an increase in social cohesion among citizens of the host country of the 2010 FIFA World Cup in South Africa. Similarly, Wonyoung et al. (19) found an improvement in image among residents of the city hosting the F1 Grand Prix in South Korea. Meanwhile, the social impact of small-scale sporting event has been scarce (11). For an exception, González-García et al. (20) showed that the residents of Gran Canaria supported the celebration of the Mundobasket 2014 in the island because this sporting event increased its international recognition at a socioeconomic and sociocultural level, although the costs in the every-day life was the worst negative impact. Also, Parra-Camacho et al. (21) found that the residents of the host city highlighted their participation in the sporting event and the projection of the city image as factors influencing the willingness to support future editions of the Valencia Triathlon.

However, to address a more complete assessment of impacts in sporting events, it is important to consider the opinion not only of city residents, participants, organizers, but it is also interesting to know the opinion of tourists (i.e., foreigners or non-residents of the sporting event location). Specifically, the image of the sporting event could be conditioned by certain contextual variables such as nationality, educational level, age, and occupation (e.g., the opinion of the 2002 FIFA World Cup in South Korea differed among visitors from Japan, mainland China, and the United States; see (22) or by the economic relationship one might have had with the sporting event (e.g., the group of citizens economically dependent on tourism was more supportive of Winter Olympics than another group of citizens with no relationship to the sporting event; see (23). In scientific literature there are few studies investigating the impacts of sporting events on the opinions of residents and non-residents. To highlight the study of Añó et al. (24) who found that residents of the city of Valencia (i.e., local participants) rated the celebration of the Formula 1 Grand Prix held in Valencia capital higher, both in terms of economic impact and infrastructure, than residents of the province of Valencia (foreign participants). Yamashita et al. (25) identified achievement as the most important motivational factor for revisiting the event among residents, and performance for non-residents. Thus, Vegara et al. (26) concluded that the most valued social aspect for residents of these localities was the excitement for the passing of the 2019 Vuelta a España cycling race, whereas for tourists, it was the excitement of attending the event celebration, feeling the hospitality of the residents, and showing interest in repeating the experience of attending a sporting event outside their place of residence.

Collectively, these previous studies have been more focused on large-scale events but not on small-scale ones. Consequently, the tangible effects of sporting events (e.g., economic impacts, new sports infrastructure, urban regeneration, boosting tourism and employment, etc.) have been more commonly analysed than other intangible effects (e.g., social legacies such as community pride and entertainment, etc.; see (27). As a result, there is a need to expand knowledge about the influence of possible variables affecting social support for the organization of sporting events (28). Additionally, there is a lack of studies investigating these potential social impacts withing the group of UNESCO World Heritage cities. Hence, this study covers the research gap of literature related to possible benefits and costs of celebrating a small-scale sporting event in this type of cities, not only in residents but also in non-residents of the host city. To embrace this endeavour, we used SET as the framework to estimate positive and negative perceptions of resident and non-resident attendees in a recreational gymnastic event, when responding to different questions related to social legacies.

Specifically, the objective of the study was to seek the social impact of GIMNASTRADA (year 2019) on a sample of participants according to place of residence (i.e., a group of residents of the host city vs. another group of foreign participants) with a set of items pertaining to previous studies that estimated perceptions of participants in sporting event at a social level. The choice of GIMNASTRADA 2019 (23rd edition) was motivated by it being the edition with the highest participation of gymnasts and spectators to date, in addition to being the edition prior to COVID-19 (there was no celebration of the event in 2020). Drawing by the previous evidence, we suggest that residents of the host city will give a higher score to the items of the questionnaire related to positive social impacts, than non-residents, because they will perceive more strongly the social, socioeconomic and sociocultural benefits of celebrating the GIMNASTRADA 2019 in the host city. Additionally, the residents will score less those questions of the questionnaire related to negative social impacts, compared to non-residents, because they will underestimate the negative social impacts of celebrating the gymnastic event in the host city (20, 21).

## Method

### Sample

A total of 210 participants took part in the study (Mean age M_a_ = 37.48 years; SD = 12.04). The requirements to be part of the sample were to have been physically present in the sports pavilion where GIMNASTRADA 2019 took place (i.e., to be an attendee), and to anonymously and voluntarily complete the perceived social impact questionnaire used in the present research (i.e., to be a respondent). Therefore, a procedure of convenience sampling was used, similarly to prior studies (17, 21).

Specifically, 88.10% of respondents were residents of Cáceres (*n* = 185; M_a_ = 38.10 years; SD = 11.60) and 11.90% were non-residents of Cáceres (*n* = 25; M_a_ = 35.88 years; SD = 15.05). These visitors came from other municipalities in the Extremadura region as well as from other regions of Spain and Portugal. The identity of the participants has been kept anonymous to ensure the confidentiality of their data. Participants received information regarding the objectives of the study, but not the research hypotheses. The research was conducted following the standards of the Bioethics and Biosafety Committee of the University of Extremadura.

### Instruments

The questionnaire has been the main instrument used in previous studies to investigate the perception of attendees about different impacts of sporting events (29). Accordingly, we used this instrument to evaluate the perceptions of attendees in GIMNASTRADA 2019. However, the studies exploring attendeeś opinions about sporting events have opted until now for the use of different nomenclatures or for the inclusion of different factors of perceived impacts (58). This lack of consensus in literature drove us to design *ad hoc* a questionnaire composed of 12 items, containing different statements regarding social, socio-economic and/or socio-cultural impacts that the celebration of a small-scale gymnastic event could have in the host city. To do this, the items of this questionnaire were adapted from items pertaining to questionnaires used in previous studies and, aimed to understand the social benefits and costs of sporting events (20, 21, 30). We decided to focus on investigating different social legacies (e.g., social, socio-economic, socio-cultural legacies) to better seek their influence on attendeeś perceptions about GIMNASTRADA 2019.

The list of 12 items was as follows: Item 1: Increases recognition and promotion of the city; Item 2: I am proud that GIMNASTRADA 2019 is held in Cáceres; Item 3: Generates an inconvenience in Cáceres traffic; Item 4: Poses a security risk in Cáceres; Item 5: The economic investment does not compensate for the generated benefits (social, economic); Item 6: Alters the habitual rhythm of Cáceres and harms other activities in the city; Item 7: Causes an increase in tourism in the city; Item 8: Increases the economic activity of the city; Item 9: Generates conflicts between visitors and local residents; Item 10: Deteriorates the city and its surroundings (furniture, trash, environment, noise, pollution); Item 11: Public institutions must support the celebration of GIMNASTRADA 2019; Item 12: Favors social and cultural exchange.

Specifically, half of the items were worded positively (i.e., items specifying a positive aspect of having held the gymnastic event at a social level) and the other half were formulated negatively (i.e., items stating a negative aspect of having held the gymnastic event at a social level). The presentation order of positive and negative benefits, at a social level, was randomized to avoid biases due to item presentation order. All items were rated on a numerical Likert-type scale from 1 to 5, as previously has been done in previous studies (4, 20, 21, 30).

### Variables and design

The independent variable of the study was the *Normal place of residence of the participants* who attended GIMNASTRADA 2019 (Level 1: Residents of the city of Cáceres; Level 2: Non-residents of the city of Cáceres). The study variable was the participants' perception regarding certain social effects that GIMNASTRADA 2019 could generate in the city of Cáceres. Specifically, the participants’ degree of agreement or disagreement was quantified in relation to possible positive impacts the gymnastic event could have had regarding social and cultural exchange, increase in tourism and economic activity in the city, increase in city recognition and promotion, and pride that the sporting event is held in the city. Their degree of agreement or disagreement was also quantified in relation to possible negative impacts the sporting event could have had, such as deterioration of the city and its environment, traffic inconveniences, security risks, and conflicts between residents and non-residents.

All dependent variables had to be scored within 5-point scale (i.e., each item must be responded with an option of value matched between 1 and 5). Their meaning was as follows: (5) Participants strongly agreed with the statement of the item, (4) Participants agreed with the statement of the item, (3) Participants neither agreed nor disagreed with the statement of the item, (2) Participants disagreed with the statement of the item, (1) Participants strongly disagreed with the statement of the item. For example, if the response to the item “GIMNASTRADA 2019 promotes social and cultural exchange” achieves a mean of 4.5, it means that participants have an opinion between strongly agreed and agreed with that statement.

A cross-sectional design was used to target the research statement. This type of design, together with the use of questionnaire as instrument, has been mainly used as the usual methodological approach in most studies to analyze impacts of sporting event (for a more detail; see the meta-analysis of (29).

### Procedure

**Figure 1:**
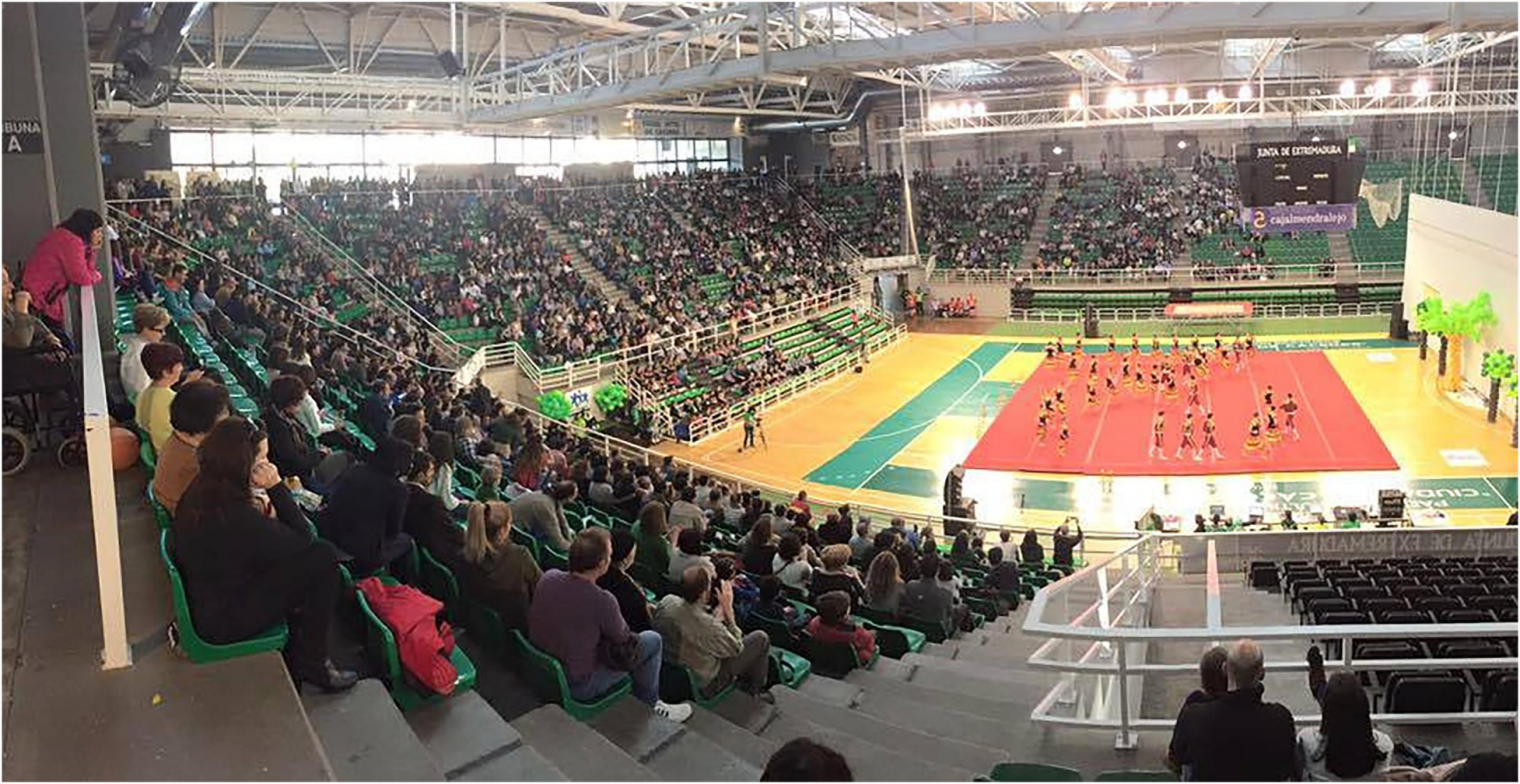
A group of gymnastics performing a choreography while the spectators observe it during the GIMNASTRADA.

First, the research team met one month before the celebration of (7) (late March) to develop the study questionnaire. On the two days of the event (March 30 and 31), all attendees of the gymnastic event were invited and encouraged to respond to the questionnaire. The technical manager of each gymnastics group was responsible for distributing the link to the questionnaire via emails and/or WhatsApp groups. Spectators of GIMNASTRADA 2019 could complete the questionnaire via a QR code available on the giant video-projection screen used to facilitate visual monitoring of the gymnastics performances (see Figure 1).

Participants had to respond individually and via their available mobile devices (e.g., mobile phone, tablet, laptop) to the questionnaire items. The Google Docs application was used to facilitate form completion. Survey submissions with participant responses were stored in an Excel file, which was available on Google Drive for consultation and subsequent analysis by the research team.

### Data analysis

First, descriptive statistics were requested based on the *Normal place of residence of the participants*. The Kolmogorov–Smirnov normality test determined that the dependent variables did not present a normal distribution of data because they were ordinal variables (i.e., their values were ranked from 1 to 5, with a meaningful order shown at the variable section). Therefore, non-parametric analyses were performed, assuming the non-distribution of data and using rank-based methods. These analyses are particularly useful with small samples of participants and ordinal variables because they are less sensitive to sample size and outliers and due to the distances between values are not necessarily equal, respectively. Specifically, the Mann–Whitney *U*-test was requested to determine if there were differences in study variables between Residents vs. Non-residents of the host city because it is the non-parametric alternative to the Student *t*-test when assumptions of normality cannot be met (e.g., independence of observations, skewed distributions, heterogeneity of variances). According to Corder and Foreman (31), this non-parametric test would be useful when data are ordinal, ranked, not normal, or when sample sizes are small as occurs in this study. The magnitude of the differences between the two groups was assessed by calculating the effect size (r) and using the formula: r_pb_ = z/√N; where z is the standardized statistic obtained from the Mann–Whitney *U*-test and N is the total sample of respondents. To interpret the practical value of this effect size, the following convention was used: 0.10: small, 0.30: medium, and 0.50: large (32).

A total of 2,520 responses were analyzed (210 participants × 12 questionnaire items). The statistical analyses included the *weight cases* command since the number of existing cases in each participant group was different. Weighting was used in this study to avoid a misrepresentation of the parameter estimates when proportions of the groups differ from the target population (i.e., weighting was used to adjust the influence of observations in each group so that the sample better reflects the population distribution). Thus, we clarify that the analyses were conducted at the item level because the objective of the study was to compare responses to individual items between residents and non-residents rather than to perform scale validation or construct-level modeling. For this reason, each comparison corresponded to a predefined variable of interest that was compressed into a statement associated with different impacts of the event. Nevertheless, to account for multiple testing across the 12 comparisons, *p*-values were adjusted using the Benjamini–Hochberg false discovery rate (FDR) procedure, with a significance threshold of 0.05. Statistical analysis was performed with the SPSS 25.0 statistical package (IBM Corp © 2017).

## Results

First, to note that the results of the Kolmogorov–Smirnov test performed in each item of the questionnaire indicated that the distribution significantly differed from normality (*p* < 0.01), both for the residents and non-residents. Second, it should be highlighted that the resident and non-resident groups participating in the study had mean values greater than 4 out of 5 points in those items stating possible positive impacts of holding GIMNASTRADA 2019 in the city (Items 1, 2, 7, 8, 11, 12). In contrast, items stating possible negative impacts of the gymnastic event in the city (Items 3, 4, 5, 6, 9, 10) obtained a mean value of less than 2 out of 5 points in both groups, except for item 5 with values slightly above 2. All items of the questionnaire yielded a small ES (minor to 0.10), except two items (Item 2: “I am proud that GIMNASTRADA 2019 is held in Cáceres”; Item 5: “The economic investment does not compensate for the generated benefits (social, economic)” that reached a small-medium ES (between 0.10–0.30) (see Table 1).

**Table 1:** Statistics for the study variables according to the based on the normal place of residence of attendees.

RESIDENTS				NON-RESIDENTS					
	D	MV (SD)	MR	D	MV (SD)	MR	U	p	r
Item 1	0.41	4.44 (1.09)	116.51	0.34	4.29 (1.23)	106.98	3,959	0.27	0.08
Item 2	0.51	4.62 (1.04)	121.11	0.35	4.33 (1.09)	96.52	3,457	0.0009*	0.23
Item 3	0.32	1.68 (0.99)	115.10	0.33	1.62 (0.77)	117	4,296	0.84	0.01
Item 4	0.40	1.42 (0.86)	114.39	0.43	1.33 (0.56)	114.90	4,301	0.95	0.00
Item 5	0.27	2 (1.25)	110.59	0.27	2.37 (1.49)	129.15	3,617	0.07	0.13
Item 6	0.39	1.45 (0.86)	114.87	0.41	1.42 (0.65)	117.88	4,254	0.73	0.02
Item 7	0.31	4.20 (1.18)	112.72	0.35	4.37 (1.06)	121.17	4,000	0.37	0.06
Item 8	0.25	4.14 (1.11)	115	0.27	4.08 (1.25)	115	4,344	0.99	0.00
Item 9	0.43	1.38 (0.80)	115.06	0.40	1.50 (1.02)	119.58	4,220	0.59	0.04
Item 10	0.39	1.46 (0.88)	113.49	0.37	1.58 (0.88)	123.10	4,003	0.28	0.07
Item 11	0.48	4.63 (0.99)	114.84	0.52	4.54 (1.25)	118	4,248	0.64	0.03
Item 12	0.44	4.57 (0.98)	116.04	0.42	4.54 (1.02)	113.47	4,270	0.74	0.02

Values for: D, Kolmogorov Smirnov test; MV, Mean value; SD, Standard deviation; MR, Mean rank; U, Mann Whitney test; p, probability value; r, effect size.

* *p* < 0,001.

To highlight that the residents showed higher mean ranks for half of items related to the positive impacts (Items 1, 2, 12), and lower scores for all items associated to the negative effects (Items 3, 4, 5, 6, 9, 10), compared to the non-residents. However, these differences were not statistically significant except for item 2. In this specific item, the residents displayed higher mean rank than non-residents (*U* = 5,653.50; *p* < .001). Also, after correction, this comparison between groups of respondents remained statistically significant (FDR-adjusted *p* < 0.05).

## Discussion

The objective of the study was to determine if the participants' place of residence is an influential variable in the perception of social impacts that a small-scale sporting event like GIMNASTRADA 2019 might have. The sporting event was held in Cáceres, a monumental city declared a World Heritage Site by UNESCO. Therefore, the analysis of the social impact of this sporting event on the city is pertinent, as social and cultural sustainability appears in the sustainable development goals of the United Nations 2030 Agenda (33). One of the most relevant findings of the study was the social impact of GIMNASTRADA 2019, in both the resident and non-resident groups, considering the high scores gave by participants to questions regarding positive impacts. These data reinforce the sporting event as an opportunity to foster social and cultural exchange, city recognition, and interaction with people from other localities, resulting in a strengthening of the “social fabric”. Furthermore, scores for negative impacts (e.g., noise, dirt, parking problems, etc.) obtained low mean values, indicating that the event did not worsen the daily quality of life of city residents. Thus, GIMNASTRADA 2019 demonstrated a certain level of urban friendliness by taking place in existing sport infrastructure, without requiring exclusion zones or extreme security.

We believe that the playful, recreational, and non-competitive character of this small sporting event, which prioritizes participation over results of gymnastic performances, limited the negative social impacts associated with mega-events (34, 35). For example, economic costs or traffic problems (19); tax increases to pay for large infrastructures to be built (23); as well as inconveniences of long trips, price increases, security or pollution problems among others (36). With these data in mind, we state that GIMNASTRADA 2019 maximized the intangible social benefits typical of a small-scale sporting event (e.g., improvement in quality of life, pride in being a resident of the host city, feeling of belonging to the local community, new opportunities for leisure and resident participation; see (21). In this line, González-García et al. (37) pointed out that the satisfaction with the event was the main variable predicting future intentions to participate in sustainable small sporting events. These results are consistent with the systematic literature review and meta-analysis conducted by Chen et al. (13), who stated that residents perceived a higher quality of life in their support for small events compared to mega-events.

Contrary to our initial expectations, the scores of items between groups of respondents were not statistically different, except for the item related to the pride of hosting GIMNASTRADA 2019 (Item 2). It is true that residents displayed higher values than non-residents for some items stating positive impacts of holding GIMNASTRADA 2019 in the city and lower values for those items stating possible negative impacts of the gymnastic event, but not to a significant level. In this vein, the effect size was small-medium for item 2, indicating a moderate practical difference in real-world terms. According to these statistics, we suggest that the practical impact of these differences on respondents' perceptions was limited except for the item 2 that showed significantly higher scores for residents compared to non-residents. This result matches with previous studies stating that residents had a greater feeling of pride that the sporting event took place in their city (14, 17, 38). Similarly, Reimers et al. (9) concluded that civic pride was one of the social perceptions influencing residents' overall attitude towards a Formula One event. Again, the effect size was between small-medium for the item 5. However, this result should be treated with caution because these differences between groups were not significantly different. For the rest of items, the differences between groups were minor. Specifically, the residents showed lower scores, compared to non-residents, when responded to the item 5. This data means that the attendees from the host city estimated more the social and economic benefits than the costs of organizing the gymnastic event than the non-residents. Again, this result is in line with previous evidences that assert a supporting to sporting events within residents of the host cities (11, 12).

Collectively, an explanation for these findings would be found within the Social Exchange Theory (39). According to this theory, residents would be willing to share certain city resources (e.g., public space, municipal funds, etc.) if the perceived social benefits (e.g., increased civic pride, cultural exchange, greater entertainment offer) are greater than the negative costs [e.g., noise, traffic, insecurity, etc.; see (14, 15, 17)]. For example, Gibson et al. (18) found greater social cohesion among residents of the host country of the 2010 FIFA World Cup in South Africa. Similarly, Wonyoung et al. (19) showed that the host community obtained the highest scores regarding social perception of the Korean F1 Grand Prix. Also, Duan et al. (11) concluded that social and psychological impacts positively predicted support and quality of life among local residents. In this line, residents understand the need to project a good image during the sporting event in order to attract tourists. In fact, a positive social perception by residents (i.e., “being good hosts”) improves the sporting tourist's experience, incentivizing repeat visits and spending (12) as well as creating a positive atmosphere at the event (25, 38). Therefore, creating a good “city-brand” image warrants special attention for event host cities and their residents because it could stimulate the arrival of new tourists, factories, companies, and talented individuals (40).

Considering the results obtained, we can conclude that GIMNASTRADA 2019 reinforced the image of the city of Cáceres, a UNESCO World Heritage City, as it had a positive social impact among visitors to the sporting event and city residents, without compromising their quality of life during the days the event lasted. It is recommended that Public Administrations maintain support for this “Sport for All” model, as opposed to the exclusive pursuit of elite competitions, since these small-sized sporting events could report multiple benefits at a social level; for example, improving the social cohesion of a city's inhabitants or cultural exchange between residents and non-visitors.

### Research perspectives

In the future, it would be interesting to include new formulas for analyzing the social impact of GIMNASTRADA. For example, through live broadcasting or *streaming*, one could assess the interest said gymnastic event has awakened among those people who, for different reasons, could not attend the sporting spectacle in person. One formula would be to quantify the number of views and/or downloads that occurred during the duration of the event, which time slots had the greatest dissemination of content in real-time via the internet, etc. In this line, it would also be interesting to know the flow of information (i.e., number of conversations, files, images, etc.) that different social media groups linked or related to GIMNASTRADA have exchanged before, during, and after the celebration of the sporting event.

In future editions of GIMNASTRADA, the social impact of the sporting event could be analyzed with a broader temporal perspective, and not only during the days of celebration, because previous studies have found that participants' opinions varied depending on whether they responded before, during, or after the completion of the sporting event. For example, Vegara et al. (26) determined that social perception after the event was slightly lower than prior to it regarding issues such as increased investment in the Municipality, benefits for social businesses, and promotion of tourism in the Municipality. Balduck et al. (41) concluded that the most valued positive aspects, prior to and after the sporting event, had to do with social and cultural recognition and perception associated with the image of the city in which the event takes place. Also, Karadakis and Kaplanidou (42) concluded that environmental legacies were the best-rated social aspects once the Winter Olympic Games concluded.

Finally, future editions of GIMNASTRADA could include an analysis of its environmental impact, as this sporting event is held in a UNESCO World Heritage City. For example, a sporting event environmental sustainability questionnaire could be designed, based on the previous study of Morán-Gámez et al. (43), since negative consequences at an environmental level are only documented for mega-events (44–46). This questionnaire should also be aligned with the United Nations 2030 Agenda (33) and with some of the 17 sustainable development goals (e.g., Goal n°3: health and well-being, n°5: gender equality, n°10: reduced inequalities, n°11: sustainable cities and communities, n°13: climate action, n°16: peace, justice, and strong institutions).

### Strengths and limitations of the study

The main strength of the study is its originality, as it is the first study to analyze the social impact of a small-scale sporting event in a UNESCO World Heritage City. Previous studies have focused on investigating the possible social effects of holding large sporting events (e.g., Olympics, America's Cup, Tour de France, European Championships, etc.), both on people residing inside and outside the city. However, there is no prior empirical evidence on the social effects of holding smaller, non-competitive events with a marked recreational character. Therefore, the current study provides novel evidence about the application of the Social Exchange Theory (SET) to “*bottom-up*” sporting events, more focused on symbolic benefits (e.g., participation, pride and recognition), rather than tangible outcomes more prominent at mega-sporting events. Also, this study expanded to incorporate an assessment of negative costs (e.g., traffic, security risk, environmental deterioration), which are particularly relevant in small-sized Heritage Cities as Cáceres (Spain).

A limitation of the study is the small sample of participants who finally completed the questionnaire, despite it being a questionnaire of only 12 items that was easy and quick to complete, in relation to the total number of participants who attended the gymnastic event. A total of 210 attendees responded the questionnaire, similar to the 248 participants in the study of Parra-Camacho et al. (21) that tested the social impact of a participative small-scale sporting event. However, this number of participants was insufficient to generalize our results to the total population attending GIMNASTRADA 2019. It would be necessary 387 participants to achieve external validity of data in our study, using the Yamané formula: *n* = *N*/[1 + N(e^2^)]; where “*n*” is the required sample size, “*N*” is the total population size (12,000 spectators) and “e” is the preferred margin of error (5%). Specifically, *a priori* G*Power analysis (version 3.1.9.7) revealed that 53 participants would be necessary per group of attendees to achieve the threshold of 80% power, an alpha level of.05, and a medium effect size (d = 0.5), using the Mann–Whitney test (47).

Also, the participant sample did not include other interest groups such as, for example, politicians and local/regional entrepreneurs, preventing the interpretation of results in the context of “Stakeholder Theory” (48, 49). Likewise, another shortcoming of the study was that the participants only responded to the questionnaire during the celebration of the sporting event, but not before and after it. This constrained the chance of offering a more `complete picture of the attendeeś perception about the social impact, using a longer timescale (e.g., a comparison between pre-event and post-event measurements), as claimed Yamashita and Ogiso (28) in their study to address residents' long-term perceptions.

Another limitation of this study is that it focused solely on the social impacts of the sporting event, overlooking other dimensions such as economic and environmental impacts. Specifically, we did not include the analysis of the economic aspect because the gymnastic event was designed with the purpose of encouraging participation and enjoyment rather than priming economic benefits. In this line, entrance to the gymnastic event was free until the capacity of the sports pavilion was reached. The event organization also managed affordable rates with hostels, campsites, and other *low-cost* accommodations for non-resident visitors with the intention of reducing their lodging expenses. Thus, the temporal duration of the sporting event was brief (2 days, 1 night). In fact, small-scale sporting event was not associated either to relevant economic costs because there was an existing sports pavilion where GIMNASTRADA 2019 was hold. Altogether, we considered that the economic impact would not be significant in the city and accordingly no economic impacts were calculated.

Regarding the environmental aspect, this study also did not include its analysis because we also considered its impact would be low, as it used an existing sports pavilion for its celebration, with good public transport communication and being located in the city center. As a result, the event's impact through the “Triple Bottom Line” or sustainability value (i.e., a joint analysis of sociocultural, economic, and environmental impacts; see (50) could not be completed to explore the integration of cultural heritage with sporting event tourism as a promising strategy in sustainable destination management (51).

### Practical applications

The evaluation of the social impact of a sporting event can be a valuable source of information for sports managers in order to better plan the sporting event in future editions. This social character information (e.g., age, origin, educational and cultural level, tastes and motivations of participants, etc.) could be very useful for Public Administrations and sports sponsors to establish a resident and visitor profile with the intention of better designing promotional campaigns for the sporting event.

In this line, it is worth noting that policymakers have the objective of increasing physical activity among the population in their agenda, and holding small sporting events could stimulate participation in sport (35, 59). Therefore, the opportunity provided by holding this sporting event in the city should be seized to hold meetings with policymakers and inform them about the importance of investing in small sporting events, as they generate social capital and create social collaboration networks (21).

Finally, Public Administrations, together with organizers of small sporting events, should follow a strategy based on the use of their own resources (e.g., local heritage and existing sports infrastructure; see (52) to revitalize the economy and tourist arrivals while improving the quality of life of residents (e.g., strengthening sports associations in the city) and reinforcing interest in heritage (e.g., investing in accessibility or sustainability improvements).

## Data Availability

The original contributions presented in the study are included in the article; further inquiries can be directed to the corresponding author.
